# Gendered Social Perceptions of “The Poor”: Differences in Individualistic Attributions, Stereotypes, and Attitudes Toward Social Protection Policies

**DOI:** 10.1007/s11199-023-01375-9

**Published:** 2023-05-24

**Authors:** Joaquín Alcañiz-Colomer, Miguel Moya, Inmaculada Valor-Segura

**Affiliations:** 1 Department of Social Psychology, University of Granada, Campus de Cartuja, s/n, 18071 Granada, Spain; 2 Research Centre in Mind, Brain, and Behavior (CIMCYC), Granada, Spain

**Keywords:** Poverty, Gender, Attributions for poverty, Support for social protection, Gender stereotypes, Classist attitudes

## Abstract

Poverty is a phenomenon that affects men and women differently. In the current research, we examined social perceptions of poor men and women across three experiments focusing on attributions for poverty, classist attitudes, and stereotypes about poor people. In Study 1, participants from the general population (*N* = 484) made more individualistic (dispositional) attributions for men’s poverty compared to women’s poverty, blaming men more for their poverty. Participants also believed that men would manage the assistance they received from the state more poorly than women. These patterns were observed across all three studies. In Study 2 (*N* = 256), we also found that more individualistic attributions for why men were in poverty predicted more negative attitudes toward social protection policies concerning men. In Study 3 (*N* = 358), we replicated the results observed in Study 2, and found that women in poverty were described as mor communal and competent than men in poverty. We interpret these results considering the operation of traditional gender roles as well as the parallelism between stereotypes of women and poor people. Our results are relevant to the framing of the proposals by social organizations, political parties, and emancipation movements that advocate for policies and programs to address poverty.

**Supplementary Information:**

The online version contains supplementary material available at 10.1007/s11199-023-01375-9.

Poverty is a reality present in almost all societies, and Spain stands out in the European Union for its high rate of poverty and its limited capacity to reduce it: one in five Spaniards live below the poverty line (Instituto Nacional de Estadística, 2020a). As a result of the coronavirus pandemic, these poverty rates have further increased (FOESSA, 2021). There are numerous, and related, explanations for the ubiquity and persistence of poverty: economic factors (the capitalist system that guides the genesis of resources and their distribution), social factors (e.g., legal norms for the different groups that make up society), or historical reasons, among other explanations. Social psychologists have suggested that the explanations that people give about poverty, the stereotypes that exist about people in poverty, and shared ideologies about this reality can contribute to perpetuating poverty.

A wide variety of economic indicators show that poverty does not affect men and women similarly. For instance, in 2020, according to the Spanish National Statistics Institute (Instituto Nacional de Estadística [INE], 2020a), the percentage of Spanish women at risk of poverty was 21.7%, while the percentage of men was 20.2%. The percentages of women with material deprivation were greater than those for men across almost all indicators: for instance, 23.2% of women (vs. 22.4% of men) indicated that they make ends meet with difficulty or great difficulty (INE, 2020a). Most people’s main form of access to societal resources is through income derived from labor. In relation to this, in the last quarter of 2020, the percentage of unemployed women in Spain was 18.39%, compared to 14.39% of men (INE, 2020b). Moreover, in this same period, the percentage of women in temporary employment was 25.6% compared to 22.9% for men (INE, 2020b). As a final example, the number of Spanish single-parent households headed by a woman was 1,582,100 in 2020, while the number of single-parent households headed by a man was 362,700. This may be related to poverty because the percentage of 38.9% of single-parent households were at risk of poverty in 2020 (INE, 2020b).

In this research, we examine whether thinking about the gender of the person in poverty influences causal attributions about poverty, ambivalent classism, stereotypes (in terms of competence, agency, and communality), perceptions of their ability to manage support provided to them, and attitudes towards social protection. In addition, we test the mediating role of individualistic attributions in the association between perceived group in poverty (men or women) and support for social protection policies related to poverty.

## Attributions for Poverty and Gender

Poverty can be attributed to internal factors that may be viewed as controllable (e.g., lack of will) or uncontrollable factors (e.g., lack of capacity), which we refer to as *individualistic* (or dispositional) attributions. Poverty may also be attributed to external factors beyond the control of individuals (e.g., shortage of jobs; Weiner et al., 2011), which we refer to as *structural* (or situational) attributions. There are other possible causes to which people may attribute poverty, such as fatalistic (e.g., bad luck; Feagin 1972) or cultural ones (e.g., the breakdown of the nuclear family; Cozzarelli et al., 2001). We focus on the basic distinction presented above between individualistic and structural factors, since research has shown that sometimes they do not appear as a distinct factor (Cozzarelli et al., 2001; Furnham, 1982).

Causal attributions about poverty are relevant for explaining support for social protection policies related to living in poverty. Endorsement of individualistic attributions has been linked to less support for government interventions (Piff et al., 2020) and greater agreement with the idea that too much money is spent on social programs (Alston & Dean, 1972). Greater endorsement of structural attributions has been related to more positive attitudes toward people in poverty (Cozzarelli et al., 2001) and preferences for progressive welfare policies (Bullock et al., 2003).

To our knowledge, only one study has addressed the question of whether there are different patterns of causal attributions for men and women in poverty. In an exploratory study with a sample of U.S. college students, Cozzarelli et al. (2002) found that individualistic attributions for men’s poverty centered on issues related to lack of effort. These motives were also present in attributions for women’s poverty, although to a lesser extent.

Why in the case of women, in comparison with men, is it less likely that perceivers attribute poverty to individualistic causes and more likely that perceivers make structural attributions? The field of paid labor has traditionally been a male-dominated area, with the breadwinner role being the responsibility of men. Women have historically been excluded from the sphere of work outside domestic space (see Pfau-Effinger 2004). Women’s work was considered optional, not something they did of their own free will (internal causes) but when circumstances required it (due to external causes). At the same time, the research has shown that people think that both wealth and poverty are due to internal dispositions rather than external factors that are beyond one’s control (e.g., Bullock et al., 2003; Davidai, 2018). Thus, in line with the research of Cozzarelli et al. (2002) and given the higher expectations that men will dedicate themselves to work and develop a career, we think that the trend to attribute poverty to internal or individualistic causes would be stronger in the case of attributions for men’s poverty (vs. women’s poverty); and the attribution of poverty to structural causes would be stronger in the case of women’s poverty (vs. men’s poverty). Gender role expectations and norms associated with women and men could influence the way they are perceived, even in the context of poverty. From a gender-stereotyped perspective, people may be more lenient towards women in poverty compared to men, as men are typically expected to be the breadwinner and therefore, not be poor. In contrast, women are often seen as dependent on men, so if they are poor, it may be perceived as less directly their fault. In addition, previous research on the study of attributions of success and failure of men’s and women’s in the workplace showed that people make more internal attributions for men’s (compared to women’s) success in leadership roles in masculine or unspecified industries; and make more internal attributions for women’s success in leadership roles in feminine industries (García-Retamero & López-Zafra, 2009).

We propose that poverty attributions may be an important mechanism for explaining differences in social protection support depending on whether the focus is on men or women in poverty. On the one hand, previous research has shown that the perceived group of people in poverty influences causal attributions about their situation (Alcañiz-Colomer et al., 2022; Henry et al., 2004). For instance, Henry et al. (2004) demonstrated that individuals receiving welfare are subject to greater blame for their poverty compared to those who are simply described as poor. On the other hand, attributions for poverty have been causally linked to redistributive preferences. Using an experimental priming paradigm, Bai et al. (2022) found that participants exhibit reduced support for redistribution after being primed with a text passage emphasizing internal attributions (compared to those exposed to a passage emphasizing external attributions. This, together with the above explanation, leads us to propose that when perceiving a man, compared to a woman, it is the different attributional pattern about their poverty that would lead to a different attitude towards social protection. In short, we expect that participants will have more positive views of social protection policies when thinking about women compared to men, and that these target gender differences can be explained in part by the different attributions people make toward men versus women for their poverty.

## Stereotypes About Men and Women in Poverty

Two basic dimensions are understood to distinguish perceptions of other people and groups: competence (agency) and warmth (communion; Cuddy et al., 2008), and some authors have further distinguished between competence (e.g., intelligent, competent) and agency (e.g., bold, adventurous; Eagly et al., 2020). In this paper, we focus on communion, agency, and competence as stereotypical traits differentially assigned to men and women. Traditionally, communal traits (e.g., sensitivity, emotionality) are ascribed to women, whereas competence and agentic traits are ascribed to men, although these stereotypes have varied over time (Eagly et al., 2020; Moya & Moya-Garófano, 2021). For example, perceptions of competence for men and women have become more similar over time, whereas women are still perceived as having more communal and less agentic traits than men (Eagly et al., 2020).

Images and stereotypes of people with fewer resources can serve to justify and perpetuate their situations. Stereotypes about people in poverty are more negative than those about the middle class (Cozzarelli et al., 2001): those in poverty tend to be perceived as warm, but lacking competence (Durante et al., 2013, 2017). Thus, stereotypes of women and people in poverty have a certain parallelism, as both groups tend to be seen as sociable and less competent than men and upper-class people. For example, both groups tend to be similarly animalized, although for different reasons. Women are considered more emotional than men (Plant et al., 2000), and poor people are also considered more incapable of controlling their emotions (Sainz et al., 2020). In one case, they may be perceived as more dependent on men, while in the other, they may be seen as reliant on public assistance or society at large. Indeed, like ambivalent sexism, concepts have been developed to investigate hostile and paternalistic classism toward individuals from lower socioeconomic backgrounds (Jordan et al., 2021). In a social context characterized by unequal power dynamics, such stereotypes often serve to justify and perpetuate these power imbalances, ensuring that these groups remain subordinated. However, when it comes to gender stereotypes surrounding individuals living in poverty, it is not entirely clear that the same stereotypes are directly applied to both men and women. Due to the differing gender expectations and roles discussed previously, the stereotypes surrounding poverty may differ between genders.

## Attitudes Toward Social Protection Policies

Social protection policies are one way in which welfare states can protect their citizens from the inequalities and adverse effects produced by the economic system. People’s attitudes toward these policies are important because public political preferences can influence the types of policies that are implemented (Brooks & Manza, 2006; Burstein, 2003). In the case of protecting low-income individuals, these attitudes can be influenced by causal attributions for these situations (Bullock et al., 2003), stereotypes (Shepherd & Campbell, 2020), and ideologies (Blekesaune & Quadagno, 2003; Hasenfeld & Rafferty, 1989).

As a system-justifying ideology, ambivalent classism is particularly relevant to the current research. Jordan et al. (2021) proposed that ambivalent classism encompasses both hostile and benevolent beliefs. Hostile classism includes the belief that people with fewer resources should be controlled, as they are seen as insubordinate. For instance, the belief that low-income individuals should lose their benefits if they do not meet some behavioral criteria (e.g., completing necessary paperwork to receive benefits on time). Benevolent beliefs in the form of protective paternalism include the belief that people with fewer resources need care and guidance to become productive members of society. Hostile classism correlates negatively with support for progressive welfare policies and positively with restrictive welfare policies, whereas protective paternalism correlates positively with support for both (Jordan et al., 2021, Study 4). The construct of ambivalent classism mirrors that of ambivalent sexism, which includes both hostile sexist attitudes (negative views of women) and benevolent sexist attitudes (subjectively positive but stereotypical views of women in certain specific social roles; Glick & Fiske 1996). These ideologies may influence the causal explanations given for different social groups’ outcomes (Brandt & Reyna, 2011). For example, Connor and Fiske (2019), using both correlational and experimental designs, found that hostile sexism was linked with internal attributions for gender income inequality, claiming that one’s personal choices cause gender income inequality. That is, people with higher hostile sexism scores (or exposed to the condition where hostile sexism was primmed) explained the gender wage gap in terms of women’s personal choices to a greater extent. This in turn led to greater acceptance of gender income inequality. It is also possible that sexism, specifically benevolent sexism, is related to perceptions of how women in poverty manage resources.

Additionally, there are other factors that could be important in influencing attitudes toward social protection policies. Deservingness perceptions are one factor that affects the attitudes toward social assistance, referring to beliefs about whether individuals should have access to such assistance based on their personal characteristics or behavior (Appelbaum, 2001). For example, widows are generally considered more deserving of assistance than teenage mothers. Beliefs about how beneficiaries will manage benefits is another relevant factor. When individuals perceive those beneficiaries will waste resources, they show less support for welfare policies (Sainz et al., 2020). Finally, beliefs about the potential effects of different types of aid on its recipients can also be relevant to understanding attitudes towards social protection.

In relation to the different types of assistance, it is possible to distinguish between dependency-oriented assistance and autonomy-oriented assistance, which may reflect power relations between groups (Nadler, 2002). Dependency-oriented assistance seeks to provide a complete solution to the problem, with the understanding that the assisted persons cannot contribute to that solution. The second type, autonomy-oriented assistance, is focused on providing tools for the assisted persons to solve their own problems, assuming that they can do so (Nadler, 1997). These two different types of assistance have also different consequences. For instance, to maintain their privileged position, members of high-status groups may prefer to provide dependency-oriented assistance, especially when the people who will receive such aid are considered potential competitors (Nadler, 2002).

Regarding the psychological consequences for beneficiaries of dependency-oriented assistance, those who receive cash benefits experience greater autonomy than those who receive vouchers (Álvarez et al., 2018). Bearing this in mind, we explore whether differences exist in preference for support programs that promote autonomy or dependency depending on the gender of the person in poverty. Our rationale is that policies promoting autonomy may be preferred to a greater extent for groups that are considered capable of overcoming their situation. That is, if men are perceived as responsible for both earning and avoiding poverty, they may be seen as more capable of improving their situation on their own. As a result, policies that promote individual autonomy may be preferred for them.

## The Present Research

The objective of our research was to analyze the perceptions of men and women in poverty and how the gender of the person in poverty influences support for social protection policies. To do so, we focused on others’ beliefs about the causes of their poverty, the content of stereotypes regarding gender and poverty, and system-justifying ideologies related to social class and gender. All materials, measures, databases, and preregistration for our studies can be found at https://osf.io/c7qfw/?view_only=17e978b6cff640d4b06114c96fae0f77. All sociodemographic information about participants in all studies is presented in Table S1 in the Supplement A in the online supplement. This research has been conducted to conform to the American Psychological Association’s Ethical Principles in the Conduct of Research with Human Participants and studies were reviewed and approved by an institutional review board.

### Hypotheses for Study 1

We predicted that women in poverty would receive less individualistic and more structural attributions than men (Hypothesis 1). We expected that participants perceiving different groups (women in poverty vs. men in poverty) would lead to different scores in the following variables. We expected that participants would show a greater preference for autonomy-oriented policies (Hypothesis 2a) and a lower preference for dependency-oriented policies (Hypothesis 2b) for men in poverty, compared to women in poverty. Furthermore, we hypothesized that participants would exhibit more protective and paternalistic behaviors and attitudes (Hypothesis 2c), as well as fewer hostile and classist attitudes (Hypothesis 2d), towards women in poverty than towards men in poverty. We also predicted that participants would believe that a woman in poverty would manage her assistance more efficiently than a man in poverty (Hypothesis 2e). These hypotheses were preregistered.

We also preregistered several exploratory hypotheses for Study 1 regarding the mediating roles played by individualistic attributions for poverty, protective paternalism attitudes, and hostile classism in the association between one’s perception of a man or a woman in poverty and preferences for dependency-oriented or autonomy-oriented policies. We also included the Ambivalent Sexism Inventory in an exploratory test in Study 1.

### Hypotheses for Study 2

Alongside testing Hypothesis 1 again, we expected that participants would show more positive attitudes toward social protection policies when they think about a woman in poverty as the policies’ recipient, compared to those who think about a man in poverty (Hypothesis 3). We also expected that the association between the gender group and support for social protection policies would be mediated by causal attributions of poverty (Hypothesis 4): structural attributions for poverty would be greater in women than men, and individualistic attributions for poverty would be greater in men than women, and, in turn, more structural attributions will be associated with less negative attitudes toward social protection policies, whereas more individualistic attributions will be more associated with more negative attitudes toward social protection policies.

### Hypotheses for Study 3

In Study 3, we tested Hypothesis 1, 3 and 4 again. Further, we predicted that women in poverty would be perceived as having more communal traits than men (Hypothesis 5a), as less agentic than men (Hypothesis 5b), and as equally competent than men (Hypothesis 5c).

## Study 1

In the first study, we aimed to investigate whether perceptions of men and women in poverty vary in terms of individualistic and structural attributions. In addition, we also test whether attitudes toward how people manage received support and ambivalent classism (Jordan et al., 2021) vary depending on the gender of the target person in poverty.

### Method

#### Participants and Procedure

The initial sample was composed of 572 participants from the general population who completed an online survey hosted by Qualtrics (Qualtrics, Provo, UT). Data from 88 participants were removed because they met the preregistered exclusion criteria: 16 failed the attention check, 23 reported that Spanish was not their native language, and 49 completed the survey in more than one hour. The final sample was composed of 484 participants. Two-hundred forty participants answered the survey related to men in poverty (134 women, *M*_age_ = 35.50, *SD* = 14.08) and 244 answered the survey related to women in poverty (157 women, *M*_age_ = 37.99, *SD* = 15.08). We conducted sensitivity analysis for differences between two independent groups using G*Power (Version 3.1; Faul et al., 2007). This suggested that we could reasonably detect an effect size as small as *d* = 0.25 with a power of 0.80.

#### Materials and Measures

##### Gender of Poor Target Condition

Participants were randomly assigned to one of two conditions. In each condition, they had to answer a survey about a group of people in poverty. In the poor woman condition, the survey questions specifically asked about women in poverty. In the poor man condition, the survey questions specifically asked about men in poverty. Items from the survey were standardized across the conditions except for the gender of the target. We included an attention check item asking which group of people in poverty (men or women) they were thinking of when they answered the questions.

##### Poverty Attributions

We measured participants’ attributions for poverty using 20 items translated and adapted for Spanish (Furnham, 1982; Weiner et al., 2011). We also added four items concerning the Spanish context (e.g., “The seasonality of the labor market”). Participants responded to all items on a 5-point Likert scale ranging from 1 (*strongly disagree*) to 5 (*strongly agree*). Nine items were used to assess the individualistic attributions dimension (α = .80; e.g., “lack of ability,” “lack of will,” “people do not want to move to other places to work”), and 10 items were used to assess structural attributions (α = .81; e.g., “lack of access to jobs,” “bankruptcy of companies,” “low wages”). In this case, the individualistic attributions comprised both controllable and uncontrollable causes, but all of them were internal. We averaged across items for each type of attribution. Higher scores for individualistic attributions indicated greater blaming of the person living in poverty; higher scores for structural attributions indicated greater support for the idea that broad social factors were the cause of poverty.

##### Attitudes Toward Dependency- and Autonomy-Oriented Social Policies

We measured degree of agreement with 12 social protection policies, with participants using 5-point Likert scale ranging from 1 (*strongly disagree*) to 5 (*strongly agree*) to respond to items. We measured attitudes toward dependency-oriented social policies with seven items (e.g., “Give stamps to poor women (men) to exchange for basic products like food or hygiene products”) and attitudes toward autonomy-oriented social policies with 5 items (e.g., “Give cash or checks to women (men) with few resources to spend as they see fit”). We were unable to analyze data from this measure as explained in the “Results” section.

##### Ambivalent Classism

This variable was measured using the Ambivalent Classism Inventory (Jordan et al., 2021), which also uses a 5-point Likert scale (1 = *strongly disagree* to 5 = *strongly agree*), validated and adapted for Spanish by Sainz et al. (2021). We changed the term “poor people” to “men in poverty” or “women in poverty” to adapt the scale to each condition. This scale is composed of three dimensions: hostile classism (α = .89; 12 items; e.g., “By and large, if you give poor women (men) an inch, they’ll take a mile”), protective paternalism (α = .85; 4 items; e.g., “Poor women (men) ought to receive extra help with making good decisions about their health”), and complementary class differentiation (4 items; e.g., “Poor women (men) are often more humble than nonpoor people”). For our analysis, we used the hostile classism and protective paternalism dimensions to the extent that our interest was focused on the differences between the two groups and not on complementary class differentiation. Higher scores meant higher hostile classism and protective paternalism, respectively. In Spanish, the two subscales have shown good internal consistency in the past: hostile classism α’s = .93–.94, and protective paternalism α’s = .87–.89 (Sainz et al., 2021).

#### Ambivalent Sexism Inventory

Ambivalent sexism was measured using a 5-point Likert scale (1 = *strongly disagree* to 5 = *strongly agree*) validated in Spain by Expósito et al. (1998) from the original version (Glick & Fiske, 1996). This scale comprises two subscales with 11 items each: hostile sexism (α = .91; e.g., “When women lose to men in a fair competition, they typically complain about being discriminated against”) and benevolent sexism (α = .83; e.g., “Women should be cherished and protected by men”). Mean scores were used, with higher scores representing higher levels of sexism. The structural validity of scores for the Spanish version of the Ambivalent Sexism Inventory has been supported via both exploratory and confirmatory factor analyses (Expósito et al., 1998).

##### Perceived Use of Assistance

We included three items on a 5-point Likert scale (1 = *strongly disagree* to 5 = *strongly agree*) to measure perceptions of poor women’s and men’s use of assistance provided to them (α = .76). The items were “Women (men) with few resources will make good use of the aid they receive;” “Women (men) with few resources will waste part of the aid they receive;” and “Women (men) with few resources will adequately manage the aid they receive.” Although it is probable that the participants were thinking of financial assistance, given the context and phrasing, we used the term ‘aid’ generically without specifying the type of assistance. We averaged scores across items. Higher scores indicated greater perceived efficiency in using assistance. Detailed distributions for these variables across conditions are presented in Supplement B1 in the online supplement.

##### Political Ideology

Political ideology was assessed by asking participants to place themselves on a scale ranging from 1 (*extreme left*) to 10 (*extreme right*).

##### Objective and Subjective Socioeconomic Status

To measure objective socioeconomic status, participants indicated their income and education levels. We standardized and summed these scores (see Piff et al., 2010). We measured subjective socioeconomic status using the MacArthur Scale of Subjective SES, a ladder with 10 rungs representing higher levels of education, income, and occupational status at the top of the ladder, and lower levels at the bottom. Participants placed themselves on the rung where they felt they stood relative to society.

###### Gender and Age

Participants also indicated their gender (man/woman/other) and age.

### Results

#### Preliminary Analyses

As preregistered, we conducted a factorial analysis of participants’ attitudes toward dependency- and autonomy-oriented social measures. This analysis revealed a three-dimensional factor structure. These dimensions were difficult to interpret at a theoretical level, and the reliability indices of their subdimensions were low. Given this, we decided not to include these variables in subsequent analyses despite having preregistered them. Consequently, we did not analyze the hypotheses concerning these variables (Hypothesis 2a and 2b).

To interpret the effect sizes we followed the benchmarks in social psychology established by Lovakov and Agadullina (2021), which rely on empirically derived effect size distributions different from those suggested by Cohen (1988). Lovakov and Agadullina suggest interpreting effect sizes (in Cohen’s *d*s) of 0.15, 0.36, and 0.65 as small, medium, and large effects, respectively.

#### Hypotheses Testing

We performed a *t*-test for differences between two independent means using SPSS (Version 25). Hypothesis 1a predicted that women in poverty would receive less individualistic attributions and more structural attributions than men in poverty. As shown in Table 1, this hypothesis was partially confirmed: women in poverty received less individualistic attributions in comparison to men in poverty, *t*(482) = 5.23, *p* < .001, Bca 95% CI [0.23, 0.50], *d* = 0.48. However, we did not find statistically significant differences in structural attributions between participants who were presented with a man in poverty and participants who were presented with a women in poverty, *t*(482) = 0.29, *p* = .78, Bca 95% CI [-0.11, 0.15], *d* = 0.03.

**Table 1:** Means and Standard Deviations for Main Measures in All Studies

	Study 1		Study 2		Study 3	
	Condition		Condition		Condition	
	Men in poverty	Women in poverty	Men in poverty	Women in poverty	Men in poverty	Women in poverty
Individualistic attributions	3.09^a^ (.72)	2.72^b^ (.80)	3.22^a^ (.78)	2.89^b^ (.79)	3.27^a^ (.67)	2.96^b^ (.66)
Structural attributions	3.50^a^ (.73)	3.48^a^ (.71)	3.93^a^ (.60)	3.98^a^ (.60)		
Protective paternalism	3.46^a^ (.97)	3.15^b^ (1.12)				
Hostile classism	2.22^a^ (.73)	1.84^b^ (.75)				
Perceived use of assistance	3.41^a^ (.74)	3.89^b^ (.85)				
Attitudes toward social protection policies			3.46^a^ (.62)	3.45^a^ (.71)	3.16^a^ (.57)	3.34^b^ (.56)
Communion					3.09^a^ (.67)	3.43^b^ (.68)
Agency					2.88^a^ (.64)	2.98^a^ (.59)
Competence					2.69^a^ (.88)	2.97^b^ (.97)

*Note*. Standard deviations are presented within parentheses. Within the same study, rows with a different superscript differ at *p* < .05.

Although in the opposite predicted direction, we found a statistically significant difference in protective paternalism scores between the two conditions (Hypothesis 2c): people who answered the questionnaire about women in poverty showed less paternalistic attitudes in comparison to those who answered the questionnaire about men in poverty, *t*(482) = 3.02, *p* = .001, Bca 95% CI [0.12, 0.49], *d* = 0.29. We also found differences regarding hostile classism (Hypothesis 2d): participants showed less hostile classist attitudes toward women in poverty compared to their attitudes toward men in poverty, *t*(482) = 5.65, *p* < .001, Bca 95% CI [.25, .51], *d* = .51. A similar pattern emerged in regard to perceived use of assistance (Hypothesis 2e): women in poverty were perceived to use assistance more efficiently than men in poverty, *t*(482) = − 6.62, *p* < .001, Bca 95% CI [-0.62, -0.33], *d* = 0.60. The interaction between participant gender and experimental condition did not significantly affect the scores in these variables (see Supplement B2 in the online supplement).

Although it was not preregistered, we performed a mediation analysis using the PROCESS Macro (Model 4) for SPSS (Hayes, 2017) with confidence intervals for indirect effects based on 10,000 bootstrapped samples. We included individualistic attributions as a mediator, perceived group as the independent variable, and perceived use of assistance as the dependent variable. As poverty attributions are known to influence attitudes towards social protection, we sought to determine if they also affected the perceived effectiveness of welfare use. We reasoned that if women in poverty, compared to men, receive less individualistic attributions (e.g., lack of ability), this might lead to the belief that they manage assistance better. Indeed, the gender of the target in poverty had an effect on perceived use of assistance through individualistic attributions. The indirect effect in this model was *b* = .13, Bca CI [.07, .19]. The direct effect of poor target’s gender on perceived use of assistance was *b* = .37, *p* < .001, and the total effect *b* = .48, *p* < .001. The effect of perceived condition on individualistic attributions was *b* = -.36, *p* < .001, and the effect of individualistic attributions on perceived efficacy of aid management was *b* = -.35, *p* < .001. However, when structural attributions were considered as mediators in our other analyses, this variable did not play a significant role in the relationship between perceived group and perceived aid management efficiency. In addition, we also performed several analyses exploring the moderator effects of hostile and benevolent sexism on protective paternalism and hostile classism (see Supplements B3-B7 in the online supplement).

As the correlations in Table 2 show, participants’ benevolent sexism scores were negatively correlated with the perceived use of assistance in both gender target conditions. This result suggests that the more positive view of use of assistance when a woman receives aid does not seem to be due to a condescending attitude toward her. Hostile sexism was related in the same way to the perceived use of assistance for both gender conditions, although the correlation was of greater magnitude when the target was women in poverty. Surprisingly, in the condition where the target group was men in poverty, the structural attributions correlated positively with the individualistic attributions, although this did not occur in the women target group.

**Table 2:** Correlations Between Main Variables in Study 1

	1	2	3	4	5	6	7
1. Individualistic attributions	-	.1	.37^**^	.55^**^	.33^**^	.51^**^	− .40^**^
2. Structural attributions	.47^**^	-	.01	− .12	.17^**^	− .05	.14^*^
3. Benevolent sexism	.36^**^	.11	-	.58^**^	.30^**^	.46^**^	− .17^**^
4. Hostile sexism	.45^**^	.04	.56^**^	-	.28^**^	.54^**^	− .33^**^
5. Protective paternalism	.31^**^	.32^**^	.10	.07	-	.47^**^	− .23^**^
6. Hostile classism	.48^**^	.06	.46^**^	.47^**^	.34^**^	-	− .54^**^
7. Perceived use of assistance	− .24^**^	.05	− .15^*^	− .23^**^	.02	− .44^**^	-

*Note*. Correlations between variables in women in poverty condition (above the diagonal) and men in poverty condition (below the diagonal). ** *p* < .01 (2-tailed) * *p* < .05 (2-tailed).

### Discussion

These results confirmed our hypotheses, showing a gendered pattern of attributions for poverty. Compared with women in poverty, men in poverty were perceived as more responsible for their poverty, which led to more negative views of how they use assistance. Regarding structural attributions, at least with the measure used, we found no differences between the two subgroups, although as expected given the context of our study, scores for structural attributions were higher than those for individualistic attributions (e.g., Lepianka et al., 2010). Perhaps this greater consensus regarding structural attributions makes them less of a decisive factor in the perceptions of these two groups, and less important for explaining attitudes toward social protection policies.

The participants also showed more hostile classist and protective paternalist attitudes toward men in poverty compared to women in poverty. Men in poverty were perceived as more needful of dominative control and paternalistic assistance compared with women in poverty. This makes sense given that men are typically seen as more responsible for their financial situations and less competent to manage the financial support they receive. However, it is possible that the negative view of men who are perceived as unable to ‘win their bread’ may be even greater, despite the assumption that men are expected to fulfill this role.

Finally, the correlations of the scores for sexism, both hostile and benevolent, with the other variables showed similar patterns whether it was the man or the woman in poverty: the more sexist the participants were, the more individualistic their attributions were, and the less they considered the man or woman able to handle the help well. This suggests that sexist ideology does not seem to play a differentiated role in gendered perceptions of poverty.

## Study 2

In Study 1, the main dependent variable was a simple measure (three items) about how people think that people in poverty manage the financial help they receive. Here, we included a broader measure of attitudes toward social protection policies to test whether people have more positive attitudes toward these policies when they think about women in poverty in comparison with men in poverty. We also explored how attributions for poverty may affect this pattern. In our analyses, we controlled for participants’ political ideology, gender, and subjective and objective socioeconomic status.

### Method

#### Participants and Procedure

The initial sample for Study 2 consisted of 304 undergraduate students, who completed an online survey hosted in Qualtrics (Qualtrics, Provo, UT). The participants were recruited through the mailing list of a university in southern Spain. They were sent a link to the study in the email. As compensation for their participation, they were entered into a 50 Euro raffle. Data from 48 participants were removed because they met the exclusion criteria: 21 participants failed the attention check, 20 reported that Spanish was not their native language, and seven completed the survey over periods of time that were more than three standard deviations above the mean. Of the remaining 256 participants, 124 (89 women; *M*_age_ = 22.74, *SD* = 3.79) answered a version of the survey that referred to men in poverty, and 132 (97 woman; *M*_age_ = 22.98, *SD* = 5.86) answered a version of the survey that referred to women in poverty. We conducted sensitivity analyses using G*Power (Version 3.1; Faul et al., 2007) for differences between two independent groups. Considering our sample size in each condition, the sensitivity analyses suggested that we could reasonably detect an effect size as small as *d* = 0.35 with a power of .80 and α = .05.

#### Materials and Measures

##### Gender of Poor Target Condition

Participants were randomly assigned to one of two conditions. At the beginning of the survey, they were asked to think in general about either a man in poverty or a woman in poverty. The items that followed did not refer to men and women, unlike Study 1. We included an attention check asking participants which group of people in poverty (men or women) they were thinking of when they answered the questionnaire. The purpose of this task was to confirm that participants had carefully read the text and considered its contents when responding to the questions.

##### Poverty Attributions

We used the same measure as in Study 1. The alpha coefficient for individualistic attributions was .83, and for structural attributions, it was .81.

##### Attitudes Toward Social Protection Policies

We used 20 statements about social protection policies (α = .89; e.g., “There is no reason for a person benefiting from social protection policies to be controlled by the authorities”) to assess participants’ general attitude toward social protection policies. Participants responded on a Likert scale ranging from 1 (*strongly disagree*) to 5 (*strongly agree*), and some items were reversed (e.g., “An extensive welfare system only fosters laziness”). Furnham (1985) inspired the scale, but items were reformulated, and others were adapted so that they measured attitudes toward social protection in general. To verify the scale structure, we conducted a confirmatory factor analysis and found that the indicators for the one-factor solution were acceptable (see Supplement C1 in the online supplement). Scale scores were created by averaging across the items. Higher scores indicated a more positive overall predisposition toward protection policies for people in poverty.

##### Political Ideology, Objective and Subjective Socioeconomic Status, Gender, and Age

The same measures were used as Study 1.

### Results

In both conditions, individualistic attributions correlated negatively with attitudes toward social protection policies; conversely, structural attributions correlated positively (see Table 3). All measures and pre-registered analyses can be consulted in Supplement C2 and C3 in the online supplement.

**Table 3:** Correlations Between Main Variables in Study 2

	1	2	3
1. Individualistic attributions	-	.11	− .51^**^
2. Structural attributions	.07	-	.40^**^
3. Attitudes toward social protection policies	− .61^**^	.37^**^	-

*Note.* Women in poverty condition (above the diagonal) and men in poverty condition (below the diagonal) correlations between variables. ** *p* < .01 (2-tailed) * *p* < .05 (2-tailed).

To test Hypotheses 1 and 3, we performed a *t*-test for differences between two independent means using SPSS. As in Study 1, we found partial support for Hypothesis 1: women in poverty received less individualistic attributions (*M* = 2.89, *SD* = .79) in comparison with men in poverty, *t*(254) = 3.39, *p* < .001, Bca 95% CI [0.14, 0.52], *d* = 0.42 (see Table 1). However, we did not find statistically significant differences in structural attributions between participants who were presented with a man in poverty and participants who were presented with a woman in poverty, *t*(254) = -.59, *p* = .55, Bca 95% CI [-0.19, 0.10], *d* = 0.08. The data in this study did not support Hypothesis 3—that participants would show more favorable attitudes toward social protection policies when they thought about a woman in poverty compared with those who thought about a man in poverty, *t*(254) = .27, *p* = .98, Bca 95% CI [-0.16, 0.17], *d* = 0.02.

To test Hypothesis 4, we performed a mediation analysis using the Hayes’ (2017) PROCESS Macro (Model 4) for SPSS. The confidence intervals for indirect effects were based on 10,000 bootstrapped samples. Individualistic attributions for poverty mediated the relationship between perceived target group (men in poverty in Condition 1, and women in poverty in Condition 2) and attitudes toward social protection policies (see Table 4). This indirect effect remained significant after we controlled for political ideology, objective and subjective socioeconomic status, and gender. However, structural attributions did not play a mediating role in the relationship between the perceived group and attitudes toward social protection policies (see Table 4).

**Table 4:** Mediational analyses of the role of individualistic and structural attributions in the relation between gender of poor target and attitudes toward social protection policies in Study 2

	Individualistic attributions			Structural attributions		
	*b* (SE)	95%CI	*p*	*b* (SE)	95%CI	*p*
Condition → Mediator						
	-.33 (.10)	[-.52, -.14]	< .001	.05 (.08)	[-.10, .20]	.554
Mediator → Attitudes toward social protection						
	-.47 (.05)	[-.56, -.38]	< .001	.43 (.07)	[.31, .56]	< .001
Total effect						
	.00 (.08)	[-.17, .16]	.978	.00 (.08)	[-.16, .16]	.003
Direct effect of gender of poor target						
	-.15 (.05)	[-.30, -.02]	.028	-.02 (.07)	[-.17, .13]	.782
Indirect effect through individualistic attributions						
	.16 (.05)	[.06, .30]	-	.02 (.03)	[-.05, .08]	-

*Note*. SE = Standard Error; CI = Confidence intervals. Condition 1 = Men in poverty, Condition 2 = Women in poverty.

Although female participants made more structural attributions and were more in favor of social protection policies, the interaction of participant gender with the target group condition was not significant for either of these variables (see Supplement C4 in the online supplement). That is, our findings were not due to women’s in-group favoritism bias.

### Discussion

The results of Study 2 confirmed a gendered pattern of attributions for poverty. Men in poverty were perceived as more responsible for their poverty, which led to worse attitudes toward social protection policies. Individualistic attributions for poverty mediated the relationship between the target group condition (men in poverty vs. women in poverty) and attitudes toward social protection policies. When the participants thought about men in poverty (compared to women in poverty), they made more individualistic attributions, which led to worsened attitudes toward social protection policies. These results fit with previous research on how poverty attributions influence attitudes toward social protection (e.g., Bullock et al., 2003). In Study 2, we confirmed that structural attributions—at least as we have measured them here—did not seem to play a relevant role for our object of study. Therefore, we did not include them in the results of the following study.

### Study 3

In addition to replicating findings from Study 1 and Study 2 in a general population sample, this study extended our knowledge about the previously observed differential social perception patterns of men and women in poverty. Specifically, we analyzed the roles of stereotypes in terms of agency, communion, and competence insofar as we know that gender-specific and class-specific differences exist in these stereotypical views. We also explored how these stereotypical perceptions of men and women in poverty could be related to attitudes toward social protection policies.

### Method

#### Participants and Procedure

We collected a total of 419 responses from an online survey hosted by Qualtrics (Qualtrics, Provo, UT). Participants were from Spain’s general population. Data from 61 participants were removed because they met the exclusion criteria: 17 failed the attention check, 23 reported that Spanish was not their native language, and 21 completed the survey in more than one hour. The final sample was composed of 358 participants: 186 answered a version of the questionnaire that referred to men in poverty (112 women, *M*_age_ = 27.93, *SD* = 12.69) and 172 answered a version referring to women in poverty (93 women, *M*_age_ = 27.77, *SD* = 12.38). Sensitivity analysis for differences between two independent groups using G*Power (Version 3.1; Faul et al., 2007) suggested that we could reasonably detect an effect size as small as *d* = 0.29 with a power of .80 and α = .05.

#### Materials and Measures

Poverty conditions and most of the measures in Study 2 were used once again. We presented conditions in the same way as in Study 1. Cronbach’s alpha was .71 for individualistic attributions for poverty. Although we used the full attributions for poverty scale from Study 1, including structural attributions, we did not hypothesize or analyze these insofar as our previous results showed that they were not a relevant variable, at least in the form in which they were operationalized here. For attitudes toward social protection policies, Cronbach alpha was .76.

In addition to these measures, we evaluated targets’ agency, communion, and competence. Competence (α = .85) was measured with five items (i.e., competent, self-confident, independent, competitive, intelligent) based on the stereotype content model (Cuddy et al., 2008). Agency was measured with seven items (α = .62; i.e., aggressive, daring, adventurous, courageous, dominant, withstand pressure well, and they don’t get nervous). Communion (α = .86) was measured with eight items. We used the items for agency and communion from Diekman and Eagly (2000), which were previously used in Spanish (Moreno-Bella et al., 2022): sensitive, affectionate, nice, gentle, sensible, understanding, kind, warmth, and caring. For these three constructs, participants responded on a Likert scale ranging from 1 (*strongly disagree*) to 5 (*strongly agree*). Specifically, participants were asked to rate the extent to which they believed women (or men) living in poverty were perceived as possessing these traits. Mean scores were used, with higher scores representing greater perceived levels of agency, communion, or competence. We measured political ideology and objective and subjective socioeconomic status with the same measures used in Studies 1 and 2.

### Results

Communion, competence, and agency did not correlate with individualist attributions or attitudes toward social protection; however, communion, competence, and agency did correlate with one another (see Table 5). All measures and pre-registered analyses are presented in Supplements D1 and D2 in the online supplement. Correlations among all variables are presented in Supplement D3 in the online supplement.

**Table 5:** Correlations between main variables in Study 3

	1	2	3	4	5
1. Individualistic attributions	-	− .55^**^	.08	.09	.12
2. Attitudes toward social protection	− .47^**^	-	− .8	− .13	− .05
3. Communion	.06	.00	-	.64^**^	.57^**^
4. Competence	− .08	.04	.62^**^	-	.77^**^
5. Agency	− .01	.02	.56^**^	.79^**^	-

*Note*. Correlations between variables in women in poverty condition (above the diagonal) and men in poverty condition (below the diagonal). ** *p* < .01 (2-tailed) * *p* < .05 (2-tailed).

#### Preliminary Analyses

First, we conducted a confirmatory factor analysis (CFA) to test the factorial structure of our measure of stereotypes. We used the MVN package (Korkmaz et al., 2014) to test for multivariate normality, and we used the lavaan package (Rosseel, 2012) to conduct the CFA, both in R (R Core Team, 2020). The indicators we achieved, using maximum likelihood estimation with robust standard errors for a three-factor structure, were acceptable, *X*^2^ = 378.547, *df* = 167, CFI = .91, TLI = .89, RMSEA = .06, SRMR = .06 (see Supplement D4 in the online supplement for more details about the CFA process as well as other indicators). We conducted this analysis because the literature on agency, communion, and the stereotype content model often treats competition and agency as similar dimensions (Cuddy et al., 2008), although some differences and other distinct classifications have been proposed (Abele et al., 2016).

#### Preregistered Analyses

As in Studies 1 and 2, we performed a *t*-test for differences between two independent means using SPSS. Replicating our findings in Studies 1 and 2, we found support for Hypothesis 1: women in poverty received less individualistic attributions in comparison with men in poverty, *t*(356) = 4.47, *p* < .001, Bca 95% CI [0.18, 0.45], *d* = 0.47 (see Table 1 for the means and standard deviations for these measures). Hypothesis 3 was supported in this study: participants who completed the survey referring to women in poverty showed more favorable attitudes toward social protection policies compared with participants who completed the survey referring to men in poverty, *t*(356) = − 2.99, *p* = .003, Bca 95% CI [-0.30, -0.06], *d* = 0.32.

Regarding differences in communion scores (Hypothesis 5a), women in poverty were perceived as being more communal compared with men in poverty, *t*(356) = -4.72, *p* < .001, Bca 95% CI [-0.48, -0.20], *d* = 0.50. We found no significant differences in the agency scores (Hypothesis 5b) between men and women in poverty, *t*(356) = -1.74, *p* = .08, Bca 95% CI [-0.24, 0.02], *d* = 0.18. Hypothesis 5c was also not confirmed: we found differences in competence between men and women in poverty, but these differences were in the opposite direction than we had hypothesized. Women in poverty were perceived as more competent than men in poverty, *t*(356) = -2.82, *p* = .005, Bca 95% CI [-0.47, -0.08], *d* = 0.30.

We performed a mediation analysis using PROCESS (Model 4) Macro (Hayes, 2017) for SPSS. The confidence intervals for indirect effects were based on 10,000 bootstrapped samples to test Hypothesis 4. As we expected, and replicating the findings in Study 2, individualistic attributions for poverty mediated the relationship between the perceived group (men in poverty in Condition 1, women in poverty in Condition 2) and attitudes toward social protection policies (see Table 6). This effect remained significant even after we controlled for political ideology, objective and subjective socioeconomic status, and gender. As in Study 2, the interaction between the participant’s gender and the experimental condition was not significant for these variables’ scores (see Supplement D5 in the online supplement).

**Table 6:** Mediational analyses of the role of individualistic attributions in the relation between gender of poor target and attitudes toward social protection policies in Study 3

	*b* (SE)	95%CI	*p*	
Condition → Individualistic attributions				
	-.31 (.07)	[-.45, -.18]	< .001	
Individualistic attributions → Attitudes toward social protection				
	-.43 (.04)	[-.51, -.35]	< .001	
Total effect				
	.18 (.06)	[.06, .30]	.003	
Direct effect of gender of poor target				
	.04 (.05)	[-.06, .15]	.411	
Indirect effect through individualistic attributions				
	.14 (.03)	[.06, .30]	-	

*Note*. SE = Standard Error; CI = Confidence intervals. Condition 1 = Men in poverty, Condition 2 = Women in poverty.

We tested whether stereotypical traits mediated the relationship between the perceived target and attitudes toward social protection policies. As expected, considering the correlations presented in Table 5, none of the three traits showed a significant mediating role in this relationship. For competence, the indirect effect was *b* = -.02, Bca CI [-.05, .00]; for communion the indirect effect was *b* = -.02, Bca CI [-.06, .01]; and for agency, the indirect effect was *b* = -.01, Bca CI [-.05, .01].

### Discussion

Consistent with the results from Studies 1 and 2, women in poverty were seen as less responsible for their situations, and the participants reported more support for social protection policies for them. Again, individualistic attributions mediated the relationship between the poverty condition (men in poverty vs. women in poverty) and attitudes toward social protection policies. That is, when the participants thought about women in poverty, they made less individualistic attributions, which led to better attitudes toward social protection policies. We found a similar pattern in Study 1, although with a relatively different dependent variable. In line with the previous literature about the stereotypes of women and men in general, women in poverty were perceived as more communal than men in poverty. Somewhat surprisingly, women in poverty were also perceived as more competent than men in poverty. This might make sense in line with other research showing how stereotype activation is context dependent (De Lemus et al., 2014), so it is possible that the stereotype varies depending on the group of men or the context in which they exist. We did not find significant differences in agency scores. These results partially fit with previous research on gender stereotypes, and they reinforced and deepened the pattern found in Study 2.

It should be noted that our studies focused on specific groups of women and men, namely those living in poverty. Gender stereotypes and roles, as well as widely held beliefs about poverty, are likely to influence the perceptions of these groups. As we suggested in the introduction, the public sphere has traditionally been inhabited by men, whereas women were relegated to the domestic sphere. This could lead one to think that the situations of men in poverty could be due to internal factors; thus, one might stereotypically perceive these men as less competent. Our results in Studies 2 and 3 suggest that the overall view of men with few resources, in terms of stereotypes and causal attributions, is more negative compared with the view of women in the same situation. However, the lack of a relationship between these stereotypical dimensions and individualistic attributions, as well as between stereotypical dimensions and attitudes toward social protection policies, suggests that these stereotypes do not seem to play an important role in the perceptions of women in poverty. It also suggests that they play a much more complex role than we have discussed here.

## General Discussion

The fundamental contribution of this article is twofold. First, we show that there are differences in perceptions of men and women in poverty (as targets) on several variables relevant both to ending poverty (e.g., poverty attributions and attitudes towards social protection) and to understanding how these groups (men and women in poverty) relate to each other (e.g., ambivalent classism and stereotype content). Second, we show the role of attributions for poverty as a causal mechanism to explain differences in support for social protection as a function of the gender of people in poverty.

Our studies provide convincing evidence that perceptions and attitudes toward people in poverty differ depending on whether the person is a man or a woman. Across three studies, we consistently showed that people made more individualistic causal attributions about men in poverty compared to women in poverty. In addition, stronger individualistic attitudes led to less support toward social protection policies when the targets were men (Studies 2 and 3), and men were perceived as using their assistance less effectively than women (Study 1). This is consistent with previous research on how people differentiate between types of people in poverty when attributing responsibility for their situations (e.g., Henry et al., 2004). It is also consistent with how attributions for poverty influence attitudes toward social policies (see for example Bullock et al., 2003; van Oorschot, 2000). Apparently, the weaker individualistic attributions toward women in poverty seem to have positive consequences, because women are seen as more deserving of social policies to alleviate their poverty. However, these less individualistic attributions to women could have a negative side: because poverty is not perceived to depend on the women themselves, it would be logical to think that they cannot do much to get out of their situations. It is important to note that this is not to suggest that women are inherently more protected from poverty or any similar assumptions. As stated in the introduction, poverty disproportionately affects women, who represent the majority of the world’s poor. Furthermore, despite an increase in women’s labor market participation and a narrowing of the income gap, significant class differences persist among women, as well as disparities in the impact of welfare policies as a function of their social class (Mandel, 2012). Thus, while women in poverty may be perceived more positively, this does not necessarily translate into improved material circumstances. The same mechanisms that perpetuate differences in power and income persist. These views shape people’s perceptions, but they do not necessarily change the structural inequalities that women and other marginalized groups face by themselves. In fact, previous research has highlighted how gender stereotypes can impact the advancement of women in their careers (Tabassum & Nayak, 2021).

As we have suggested, our findings can be understood, at least in part, as a logical corollary of the male breadwinner model and the exclusion of women from the productive sphere outside of the household (Pfau-Effinger, 2004). This model assigns different roles related to social reproduction, especially in capitalist societies, where social reproduction enables sustained capital accumulation (Fraser, 2016; Laslett & Brenner, 1989). If a traditional role for men is to provide the resources needed to sustain life, which in modern society is mainly achieved through paid work, it appears more likely that his failure to obtain these resources would be due to some inherent failure. However, by the same logic, when men are financially successful (compared with when women are), this also would be more likely attributed to their inner qualities, which is suggested by studies showing that men’s success is more likely to be attributed to internal factors, when it comes to leadership in masculine or unspecified industries (García-Retamero & López-Zafra, 2009). Indeed, this pattern is consistent with the results we obtained in Studies 1 and 2. Women in poverty were perceived as being more communal compared with men in poverty, which fits with the previous literature on gender stereotypes (e.g., Eagly et al., 2020). Women in poverty were also perceived as more competent, which did not fit with the literature on gender stereotypes, where “typical” men have generally been stereotyped as more competent (Eckes, 2002; Fiske et al., 2002). However, the situation may moderate this view (De Lemus et al., 2014). In addition, in Study 1, the participants showed more hostile classist and protective paternalist attitudes toward men in poverty compared with women in poverty; men were perceived as needing to be more closely supervised and unable to obtain or manage their own resources (Jordan et al., 2021). This is also consistent with research showing more negative attitudes exist toward people who do not meet the expectations of the social roles they should perform (e.g., Glick et al., 1997).

We argue that blaming men in poverty to a greater extent for their situations, as well as viewing them as more incompetent and in need of external control over their lives, may be due at least partly to a simmering and long-standing social dynamic. This dynamic has historically excluded women from the public and productive sphere, relegating them to a position of subordination. However, our studies also showed how this gendered division of social tasks has negative consequences for men who do not fulfil the expectations of their social role in the production process (i.e., those who are disadvantaged in terms of income). This would also match the psychological literature illustrating how the social construction of what it means to be a man, as well as its internalization, is an important factor in understanding the world as synthesized by the concept of precarious manhood (Vandello et al., 2008; Vandello & Bosson, 2013). This in turn is related to a lower inclination to confront sexual prejudice (Kroeper et al., 2014), among other issues. That is, although men benefit to a greater extent from the privileged position they occupy in the system due to their gender, they are also restricted by its gender mandates and treated accordingly. This is especially true for those who do not comply with the normative ideals of how they should fit into the relations of production. We interpret our results in this sense, but other ways of making sense of them are possible. In this series of studies, we have not tested this broad theoretical framework, as our objective was different. Thus, we merely propose this possible interpretation in a broader sociohistorical context. However, it is by no means the only possibility or the exclusive explanation for our results.

Despite the changing nature of gender stereotypes in the Spanish context over time, certain disparities persist. In a study by Moya and Moya-Garófano (2021), changes in gender stereotypes related to traits, role behaviors, occupations, and physical characteristics were analyzed using data collected in 1985 and 2018. The results indicated that while some stereotypes had shifted, the perception of women as more communal persisted. On the other hand, research has indicated that although men and women are generally perceived as more similar in terms of feminine characteristics, they still diverge significantly with regards to masculine traits (López-Zafra & García-Retamero, 2021). Although this provides a approximation of stereotypes and gender role ideologies, it is important to note that our study focuses on specific groups (e.g., those experiencing poverty) and there may not be a direct application of these stereotypes to these populations. Despite significant progress in gender equality in recent years, there are still areas related to our work where these advancements have been minimal. For instance, persistent gender inequalities can be observed in the areas of caregiving responsibilities, workforce participation, and income distribution (European Institute for Gender Equality, 2019).

### Limitations and Future Research Directions

As in any research study, certain limitations should be highlighted. A limitation of Study 2 was its undergraduate student sample, which may affect the generalizability of the results (although Studies 1 and 3 were conducted with samples drawn from the general population). Still, we found consistent evidence that both social perceptions and attitudes toward social protection were more positive when thinking about women in poverty compared to men. In Study 1, our measure for preferences for dependency- or autonomy-oriented protection policies did not work adequately. We believe that this topic is relevant for studying possible differences in the perceptions of women and men in poverty, as well as other groups of people with few resources. Thus, future research should focus on developing reliable measures for this variable. In addition, we used a general measure of attitudes toward social protection policies, not focused on specific aspects or policies. It would be important for future research to build upon these findings and further explore the potential effects on various aspects of social protection. We have solely examined perceptions of individuals identifying as women and men, thus concentrating on the gender binary. It is important that future research expands on this work to include gender groups beyond the binary.

Our research was also limited by examining poverty perceptions with a generic focus on “men” and “women” in poverty. However, other salient characteristics such as ethnicity or disability may also shape how specific groups are perceived. For example, as evidenced by prior research (Urbiola et al., 2022), there may be an interplay between ethnicity and social class that affects prejudicial attitudes and discriminatory behaviors. Future research should explore these intersections and their potential influence on poverty attributions and perceptions. Similarly, the results may also vary manipulating the type of woman in poverty. “Good” women, those that represent more what a stereotypical woman should be like (e.g., caregiver, etc.), receive more benevolent treatment whereas women who defy the gender rules are treated in a more hostile manner (Glick et al., 1997). Additionally, it is known that demonstrating a certain level of compliance and adherence to instructions can increase the perception of deservingness for those in need of help (van Oorschot, 2000). It would be valuable to investigate how perceptions of different subtypes of women may vary based on these factors.

We have framed our findings within a broader socio-historical explanation about the male breadwinner model and the exclusion of women from the productive sphere outside of the household. However, we did not test this empirically in our study. More theorizing and development of tools to capture these constructs is needed in the future to advance our understanding of poverty perception.

### Practice Implications

This research provides the first experimental evidence showing that attitudes toward social protection policies vary as a function of the gender of the target person in poverty, which has important practical implications. Popular and media images of poverty may play an important role in the perception of this phenomenon, as they can stress some aspects of poverty and ignore others (Bullock et al., 2001). For instance, when the media use images of homeless men to garner support for programs and policies aimed at combating poverty, negative stereotypes and attributions about their situation are likely to be reinforced, such as blaming them more for their situation. As a result, it is plausible that such policies may be viewed less favorably, given previous research indicating that attitudes toward homelessness are generally more negative (Cuddy et al., 2008), and our own findings which suggest that men are disproportionately held responsible for their poverty condition.

In a similar way, our research is relevant for social organizations, political parties, or emancipation movements in general, specially to the framing of their proposals for protection and social change, especially for proposals related to gender differences and economic resources. Given that poverty disproportionately affects women, and that society holds a less negative view of women experiencing poverty, it may be beneficial to propose targeted policies to improve their situation. Alternatively, general policies could be proposed with a detailed explanation of how they will specifically benefit women in poverty, as our research suggests that such policies are viewed more favorably when directed towards women. This approach recognizes that society tends to attribute less responsibility to women for their economic circumstances and generally holds a less negative attitude towards social protection for women in need.

For interventions aimed at raising awareness of poverty and encouraging collective participation to end poverty, as well as raising awareness of gender inequalities and their pernicious effects, this work may provide some helpful insights. Specifically, we highlight how these explanations of poverty, which are a sociohistorical product, relate to social roles, stereotypes, class attitudes, and ultimately to attitudes toward protection policies that influence whether they are incorporated into political priorities. For example, these interventions should focus on emphasizing the structural causes of poverty, for both men and women, to hold both groups less accountable. Furthermore, the focus should be on the origin of this differentiated gender perception: the stereotypical beliefs about gender and the associated expectations.

### Conclusion

Overall, our research shows that women and men in poverty are perceived differently. Specifically, men are viewed as more personally responsible for being poor than women. This greater individualistic attribution of responsibility for being poor, in turn, was linked to less support for social protection policies when the recipients are men. Moreover, men are perceived as less communal and less competent, in addition to being the object of hostile classist and paternalistic attitudes to a greater extent than women. Traditional gender roles typically assign caregiving and nurturing tasks to women, while breadwinning and provider roles are assigned to men. Our findings indicate that men who fail to fulfill their traditional role as “providers” receive more negative evaluations. However, it is important to note that this perception does not necessarily reflect reality, as women still tend to experience poverty at a higher rate than men.

## Supplementary Information

Below is the link to the electronic supplementary material.

ESM 1 (DOCX 340 KB)

## Data Availability

All materials, measures, databases, and preregistration forms for our studies can be found at https://osf.io/c7qfw/?view_only=17e978b6cff640d4b06114c96fae0f77.
