# Supplementary material for: Gendered Social Perceptions of “The Poor”: Differences in Individualistic Attributions, Stereotypes, and Attitudes Toward Social Protection Policies
Source: Sex Roles. 2023 May 24:1–17. Online ahead of print. doi: 10.1007/s11199-023-01375-9 (PMC10206369; doi:10.1007/s11199-023-01375-9)
Supplement: Supplementary file 1 — (DOCX 340 KB) [file 11199_2023_1375_MOESM1_ESM.docx]

Online supplement for Alcañiz-Colomer, J., Moya, M., and Valor-Segura, I. (2023). Gendered Social Perception of “the Poor”: Differences in Individualistic Attributions, Stereotypes, and Attitudes Toward Social Protection Policies. *Sex Roles*. Joaquín Alcañiz-Colomer, University of Granada. Email: [jcolomer@ugr.es](mailto:jcolomer@ugr.es)

**Supplement A: Socio-demographic participants information across studies.**

**Supplement B: Study 1 additional information.**

B1. Figure S1

B2. Controls and robustness checks

B3. All results of pre-registered analyses

B4. Figure S2

B5. Figure S3

B6. Figure S4.

B7. Figure S5

**Supplement C: Study 2 additional information**

C1. Confirmatory Factor Analysis (CFA) for Attitudes toward social protection scale

C2. All measured used in Study 2

C3. All results of pre-registered analyses

C4. Table S2.

C5. Controls and robustness checks.

**Supplement D: Study 3 additional information**

D1. All measures used in Study 3

D2. All results of pre-registered analyses

D3. Table S3

D4. Confirmatory Factor Analysis (CFA) for Stereotype Measure

D5. Controls and robustness checks

**Supplement A. Socio-demographic participants information across studies.**

**Table S1**

*Socio-demographic Participants Information Across Studies*

| Variable | Study 1 | | Study 2 | | Study 3 | |
| --- | --- | --- | --- | --- | --- | --- |
|  | n | % | n | % | n | % |
| Sex |  |  |  |  |  |  |
| Men | 190 | 39.3 | 68 | 26.6 | 152 | 42.5 |
| Women | 292 | 60.3 | 186 | 72.7 | 205 | 57.3 |
| Other | 2 | 0.4 | 2 | 0.8 | 1 | 0.3 |
| Not reported | - | - | - | - | - | - |
| Income |  |  |  |  |  |  |
| < 650 | 13 | 2.7 | 20 | 7.8 | 22 | 6.1 |
| 651-1.300 | 92 | 19 | 66 | 25.8 | 83 | 23.2 |
| 1.301-1.950 | 118 | 24.4 | 48 | 18.8 | 83 | 23.2 |
| 1.951-2.600 | 96 | 19.8 | 55 | 19.5 | 73 | 20.4 |
| 2.601-3.251 | 72 | 14.9 | 33 | 12.9 | 41 | 11.5 |
| 3.251-3.900 | 36 | 7.4 | 17 | 6.6 | 23 | 6.4 |
| 3.901-4.550 | 21 | 4.3 | 14 | 5.5 | 11 | 3.1 |
| 4.551-5.200 | 14 | 2.9 | 3 | 1.2 | 9 | 2.5 |
| 5.201-5.800 | 6 | 1.2 | 2 | 0.8 | 2 | 0.6 |
| > 5.801 | 12 | 2.5 | 2 | 0.8 | 7 | 2 |
| Not reported | 4 | 0.8 | 1 | 0.4 | 4 | 1.1 |
| Participant education |  |  |  |  |  |  |
| Primary School* | 22 | 4.5 | - | - | 4 | 1.1 |
| Secondary education* | 17 | 3.5 | - | - | 16 | 4.5 |
| Vocational training | 74 | 15.3 | 2 | 0.8 | 58 | 16.2 |
| High School/Diploma* | 41 | 8.5 | - | - | 20 | 5.6 |
| University not completed | 146 | 30.2 | 182 | 71.5 | 183 | 51.1 |
| University completed | 123 | 25.4 | 16 | 6.3 | 39 | 10.9 |
| Master degree | 42 | 8.7 | 36 | 14.1 | 26 | 7.3 |
| PhD | 13 | 2.7 | 14 | 5.5 | 5 | 1.4 |
| Not reported | 6 | 1.2 | 5 | 2 | 7 | 2 |

*Note*. *These options were not available in Study 1.

**Supplement B: Study 1 additional information.**

**Supplement B1. Figure S1**

*Distribution of the Responses in Each Variable for the two Experimental Conditions*
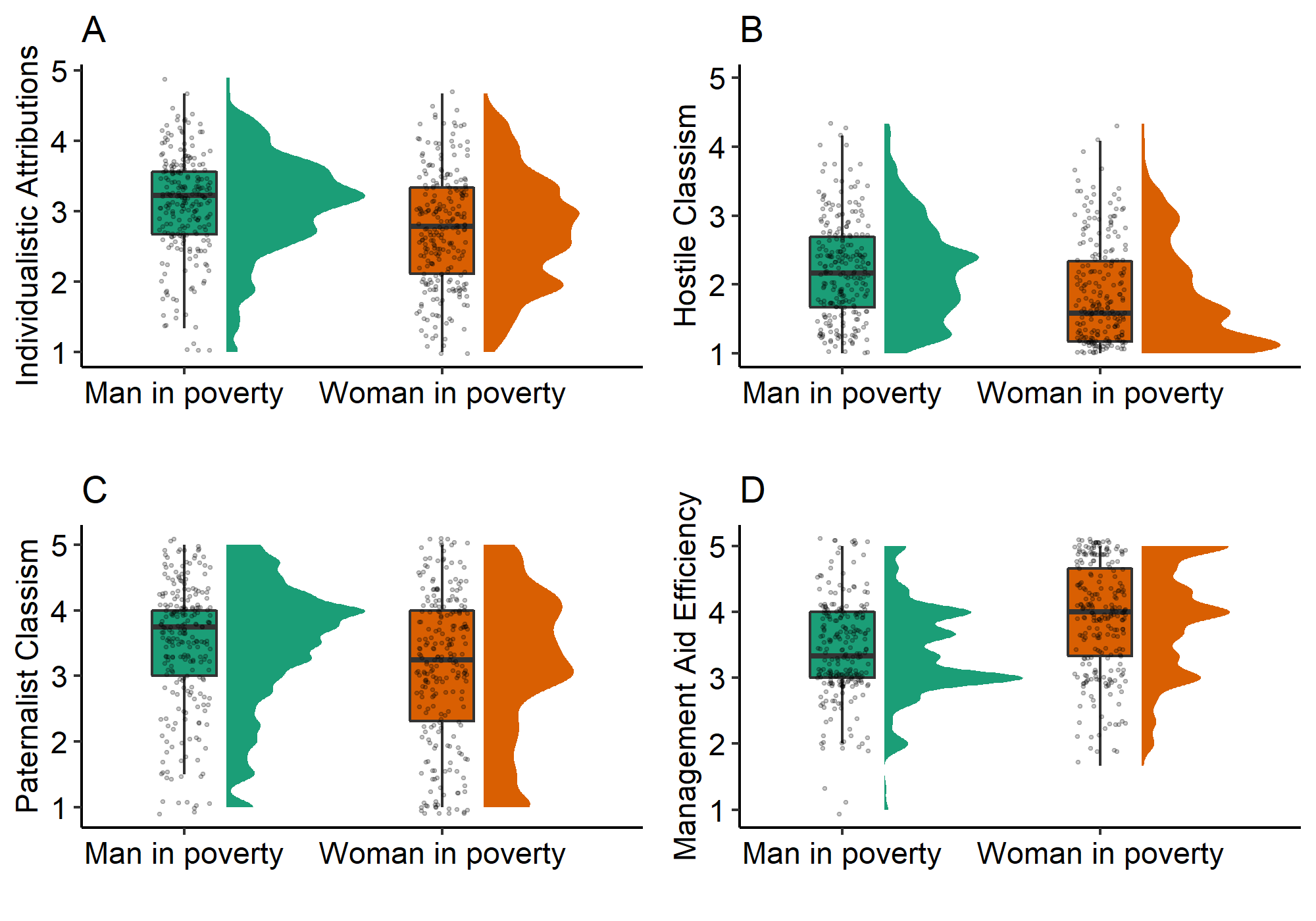


**Supplement B2. Controls and Robustness Checks**

As we state in the manuscript, we did not find a significant interaction of experimental condition with participant gender on any of our dependent variables: individualistic attributions (*F*(1, 478) = 0.08, *p* = .775), hostile classism (*F*(1, 479) = 0.05, *p* = .831), protective paternalism (*F*(1, 478) = 0.11, *p* = .737), and perceived efficiency of aid management (*F*(1, 478) = 0.52, *p* = .473).

**Supplement B3. All Results of Pre-Registered Analyses**

We performed moderation analyses including hostile and benevolent sexism as moderators, protective paternalism and hostile classism as dependent variables, and experimental condition as independent variable in separate models. Hostile sexism moderated the relation between experimental condition and protective paternalism scores *b* = 0.27, *t*(479) = 2.60, *p* = .009, 95% CI = [0.07, 0.47] (see Figure S2), but did not moderated the relation between experimental condition and hostile classism (Figure S3). On the other hand, benevolent sexism moderated the relation between experimental condition and protective paternalism scores *b* = 0.32, *t*(479) = 2.36, *p* = .018, 95% CI = [0.05, 0.57] (see Figure S4), but not with hostile classism (see Figure S5).

**
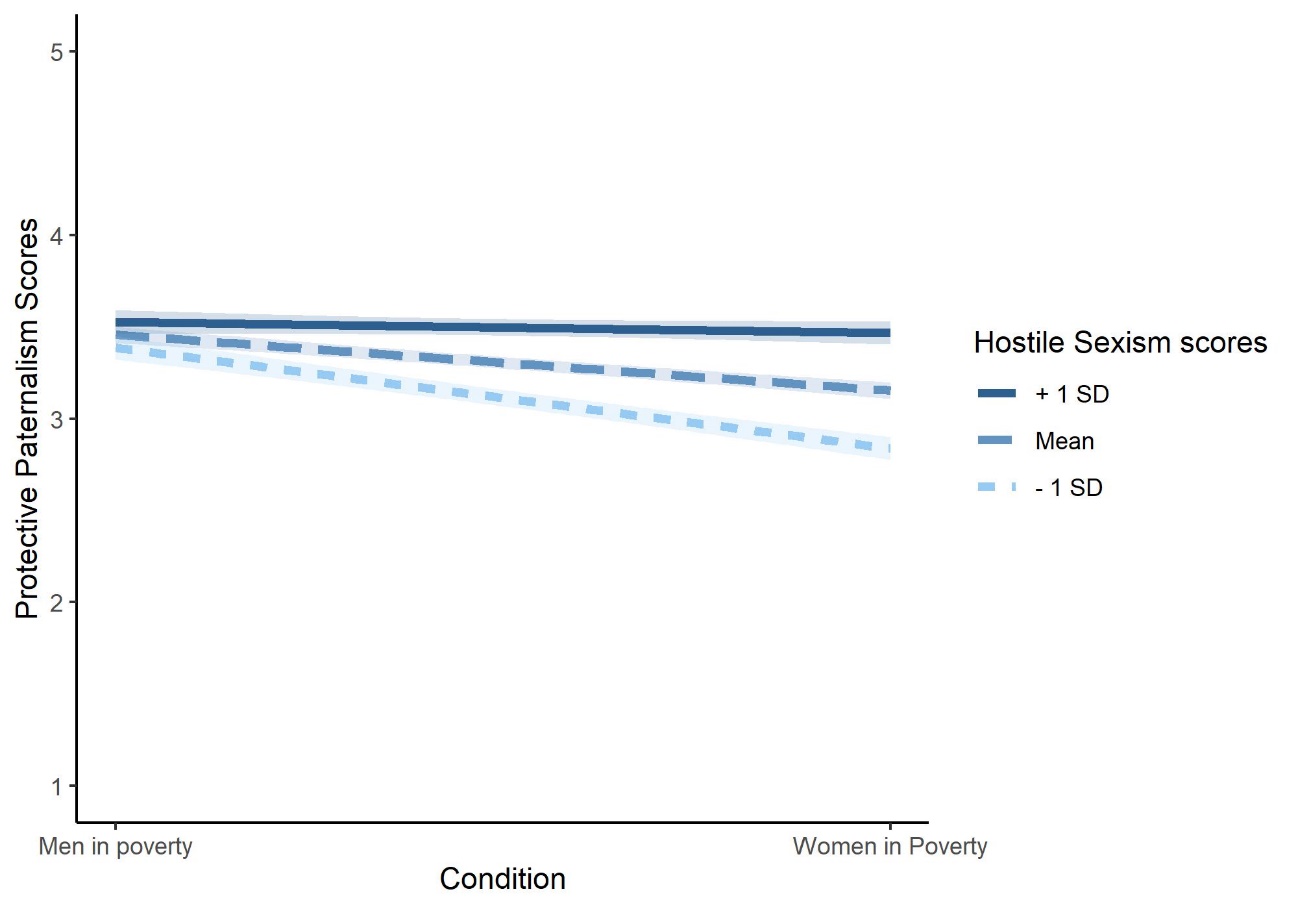
Supplement B4. Figure S2**

*Moderation Effect of Hostile Sexism on Protective Paternalism*

**Supplement B5. Figure S3**

*
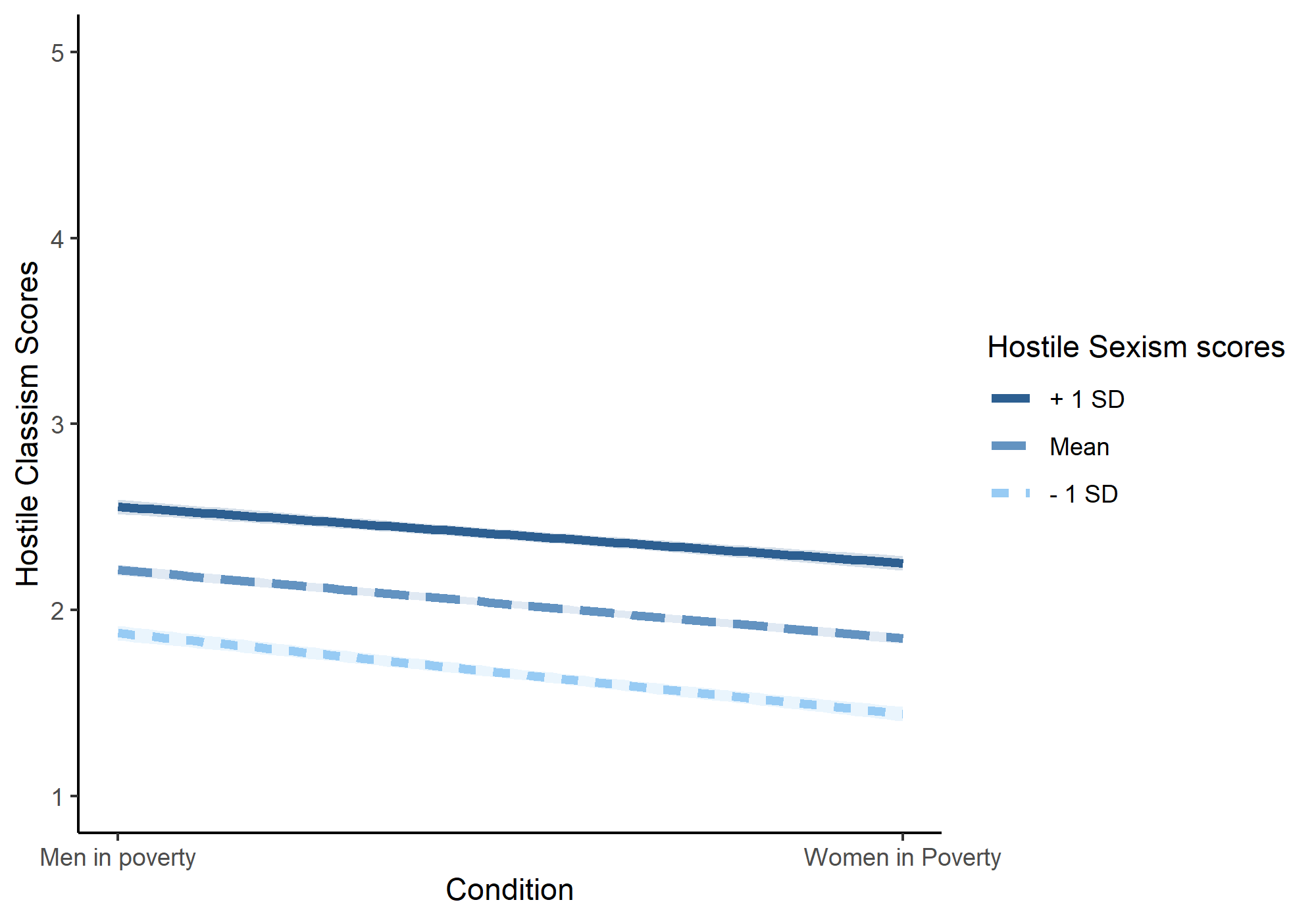
Moderation Effect of Hostile Sexism on Hostile Classism*

**Supplement B6. Figure S4**

*
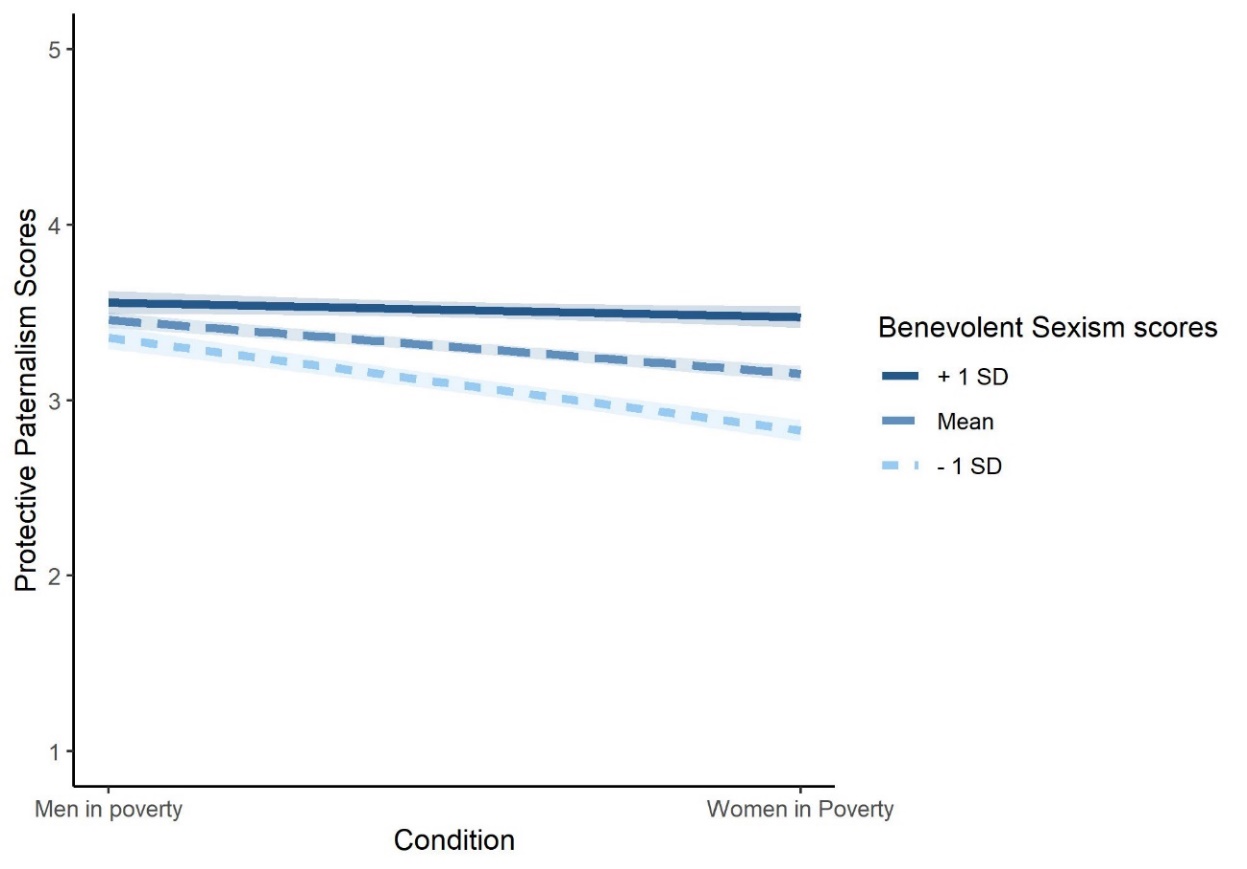
Moderation Effect of Benevolent Sexism on Protective Paternalism*

**Supplement B7. Figure S5**

*Moderation Effect of Benevolent Sexism on Hostile Classism*


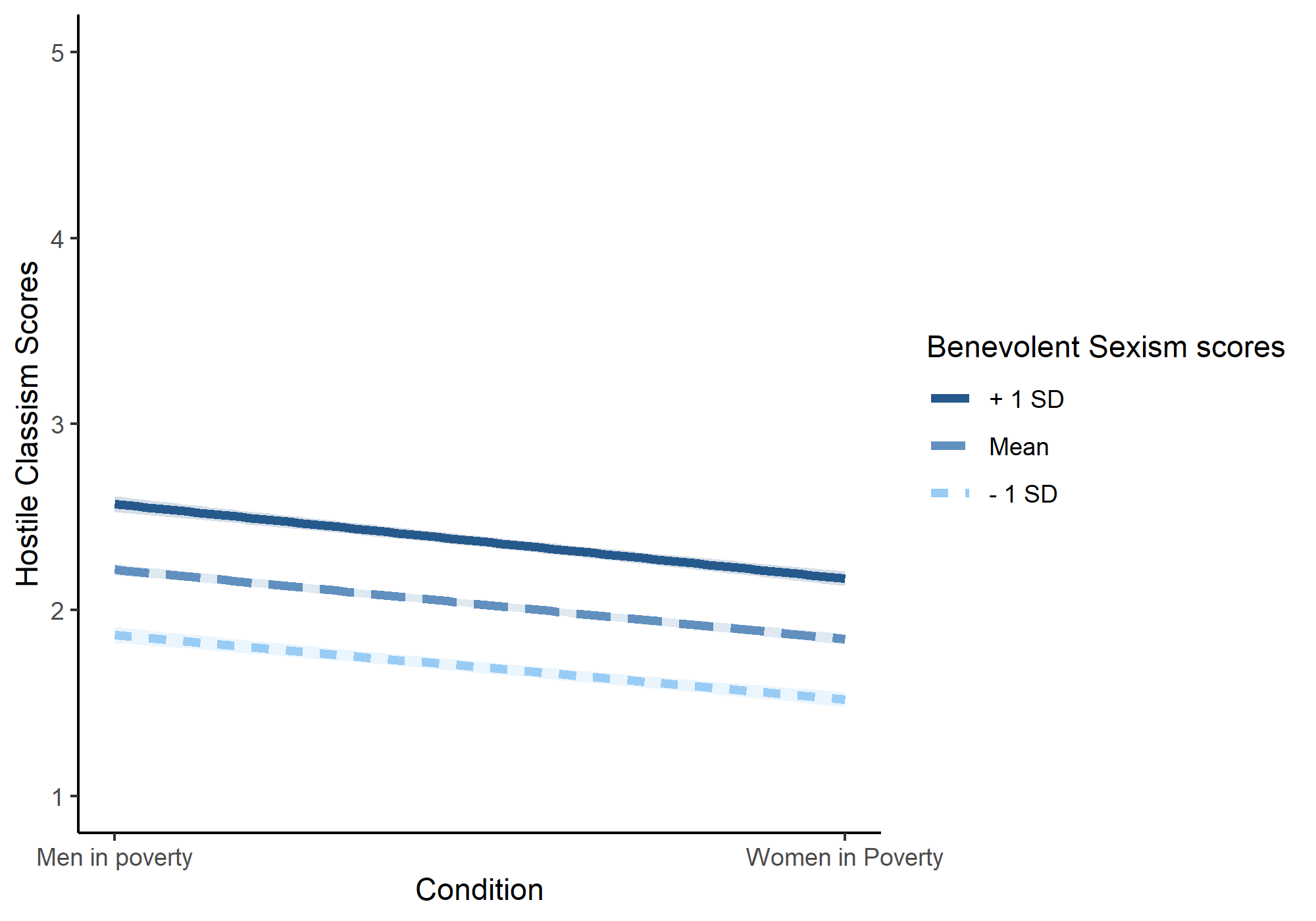


**Supplement C: Study 2 additional information**

**Supplement C1. Dimensionality of Attitudes toward social protection scale**

We used the lavaan package (Rosseel, 2012) to fit a one-factor model including all items. While the fit indicators were not poor, we further refined the model by examining modification indices and adding covariance paths between some items. This process resulted in a one-factor solution with satisfactory fit indicators *X*^2^ = 318.522; df = 165; CFI = .90; TLI = .88; RMSEA = .060; SRMR = .07.

**Supplement C2. All Measures Used**

***Precarious Manhood* *(α = .74).***

We adapted and translated to Spanish the Precarious Manhood Scale (Vandello et al., 2008; Study 1b). The scale is composed of 7 items (e.g. “Some boys do not become men, no matter how old they get”) with a 1 to 7 Likert response system. Higher scores means a higher level of precarious manhood.

***Social Dominance Orientation (α = .85)***

To measure this variable we used the adaptation and validation to Spanish version (Silván-Ferrero & Bustillos, 2007) from the original scale (Pratto et al., 1994). It consisted of 16 items (e.g., “No one group should dominate in society”) with a 1 to 7 Likert response. Higher scores mean a higher level of social dominance orientation.

**Supplement C3. All Results of Pre-Registered Analyses**

1. Favourable attitudes towards social protection policies will correlate negatively with scores in social dominance orientation scale.

There was a statistically significant relation between Social Dominance Orientation and attitudes toward social protection policies (*r* = -.59, *p* < .001).

2. Favourable attitudes towards social protection policies will correlate negatively with scores in precarious manhood.

We did not find a significant relation between these variables (*r* = -.05, *p* = .4)

3. It is expected that precarious manhood will moderate the relation between the perceived group –man in poverty (coded 0) vs woman in poverty (coded 1)- and attitude towards social protection policies.

We did not find support for this hypothesis in our data.

**Supplement C4. Table S2**

*Correlations Between Variables in Study 2*

|  | 1 | 2 | 3 | 4 | 5 | 6 | 7 | 8 |
| --- | --- | --- | --- | --- | --- | --- | --- | --- |
| 1. Individualistic attributions | - | .12 | -.50^***^ | .28^**^ | .13 | .39^***^ | .09 | -.01 |
| 2. Structural attributions | .06 | - | .40^***^ | -.40^***^ | .08 | -.22^*^ | .02 | .04 |
| 3. Attitudes toward social protection | -.61^***^ | .37^***^ | - | -.58^***^ | -.10 | -.70^***^ | -.03 | -.06 |
| 4. Social dominance orientation | .43^***^ | -.41^***^ | -.58^***^ | - | .21^*^ | .46^***^ | .14 | .15 |
| 5. Precarious Manhood | -.03 | .18 | .03 | .06 | - | -.02 | -.07 | .11 |
| 6. Political ideology | .44^***^ | -.31^***^ | -.57^***^ | .50^***^ | -.01 | - | .09 | -.01 |
| 7. Subjective Status | .03 | -.25^**^ | -.11 | .19^*^ | -.09 | .20^*^ | - | .30^***^ |
| 8. Objective Status | .18^*^ | -.07 | -.09 | .20^*^ | -.02 | .15 | .36^***^ | - |

*Note.* correlations between variables in women in poverty condition (above the diagonal) and men in poverty condition (below the diagonal). ** *p* < .01 (2-tailed) * *p* < .05 (2-tailed)

**Supplement C5. Controls and Robustness Checks**

As in Study 1, we did not find a significant interaction of experimental condition with participant gender on any of our dependent variables: individualistic attributions (*F*(1, 250) = 0.09, *p* = .469) and attitudes toward social protection policies (*F*(1, 250) = 0.53, *p* = .767).

We conducted a mediation analysis including individualistic attributions as mediator, experimental condition as independent variable, and attitudes toward social protection policies as dependent variable while controlling for gender, political ideology, subjective socioeconomic status, and objective socioeconomic status in both paths. The indirect effect was *b* = 0.08, BCa CI [0.03, 0.14]; the direct effect was *b* = -0.16, *p* = .01; and the total effect was *b* = -0.08, *p* = .23. The effect of the perceived condition on individualistic attributions was *b* = -0.27, *p* = .004, and the effect of individualistic attributions on attitudes toward social protection policies was *b* = -0.29, *p* < .001. The effect of objective status on individualistic attributions was *b* = 0.04, *p* = .31 and on attitudes toward social protection it was *b* = -0.01, *p* = .76. The effect of subjective status on individualistic attributions was *b* = -0.01, *p* = .72 and on attitudes toward social protection it was *b* = 0.02, *p* = .54. The effect of gender on individualistic attributions was *b* = -0.02, *p* = .86 and on attitudes toward social protection it was *b* = -0.10, *p* = .17. The effect of political ideology on individualistic attributions was *b* = 0.16, *p* < .001 and on attitudes toward social protection it was *b* = -0.20, *p* < .001.

**Supplement D: Study 3 additional information**

**Supplement D1. All Measures Used**

***Attitudes toward Minimum Vital Income (Ingreso Mínimo Vital)***

We measured this variable with one item (CIS, 2020), using a 1 to 5 Likert scale. Higher scores imply higher support for this measure. The heading of the question was as follows: “The Minimum Vital Income is one of the social measures taken by the government for the most needy people and sectors. We want to know if you are for or against this measure.”

***Economic impact of COVID-19 (α = .65)***

We presented six items to assess the degree to which participants think that the COVID-19 crisis is influencing Spain’s economy (2 items) and their personal economic situation (4 items), using a 1 to 5 Likert scale, ranging from “*strongly disagree*” to “*completely agree*”. Higher scores mean higher perceived economic impact.

***Impact of COVID-19 on Health (α = .75)***

We used five items to assess the degree to which participants think that the COVID-19 crisis has affected their physical and mental health using a 1 to 5 Likert scale, ranging from “*strongly disagree*” to “*completely agree*”.

**Supplement D2. All Results of Pre-Registered Analyses**

1. People who perceive women in poverty will have a better attitude toward Minimum Life Income (Ingreso Mínimo Vital, in Spanish) (in comparison to the group that perceive men).

Participants who were asked about women in poverty (*M* = 3.95, *SD* = 1.15) did not had a better attitude toward this measure in comparison to participants who were asked about men in poverty (*M* = 4.03, *SD* = 1.15), *t*(353.6) = -0.68, *p* = .50, BCa 95% CI [-0.32, 0.16], *d* = -0.07.

In addition, as in Study 2, we conducted a mediation analysis using PROCESS (Model 4) Macro (Hayes, 2017) for SPSS including individualistic attributions as mediator variable, experimental condition as independent variable, and attitudes toward social protection policies as dependent variable, controlling for gender, political ideology, subjective socioeconomic status, and objective socioeconomic status in both paths. The indirect effect was *b* = 0.13, BCa CI [0.07, 0.18]; the direct effect was *b* = 0.06, *p* = .22; and the total effect was *b* = 0.19, *p* < .001. The effect of the perceived condition on individualistic attributions was *b* = -0.34, *p* < .001, and the effect of individualistic attributions on attitudes toward social protection policies was *b* = -0.37, *p* < .001. The effect of objective status on individualistic attributions was *b* = -0.02, *p* = .44 and on attitudes toward social protection it was *b* = 0.05, *p* = .01. The effect of subjective status on individualistic attributions was *b* = 0.04, *p* = .10 and on attitudes toward social protection it was *b* = -0.02, *p* = .21. The effect of gender on individualistic attributions was *b* = 0.00, *p* = .99 and on attitudes toward social protection was *b* = 0.07, *p* = .19. The effect of political ideology on individualistic attributions was *b* = 0.07, *p* < .001 and on attitudes toward social protection it was *b* = -0.08, *p* < .001.

**Supplement D3. Table S3**

*Correlations Between all Variables in Study 3*

|  | 1 | 2 | 3 | 4 | 5 | 6 | 7 | 8 | 9 | 10 | 11 | 12 |
| --- | --- | --- | --- | --- | --- | --- | --- | --- | --- | --- | --- | --- |
| 1. Individualistic attributions | - | .15 | -.55^***^ | .09 | .09 | .14 | -.37^***^ | .01 | -.12 | .28^***^ | .19^*^ | .08 |
| 2. Structural attributions | .20^**^ | - | .23^**^ | -.20^*^ | -.17^*^ | -.03 | .19^*^ | .05 | .15 | -.29^***^ | .07 | -.03 |
| 3. Attitudes toward social protection | -.47^***^ | .29^***^ | - | -.17^*^ | -.07 | -.11 | .51^***^ | .07 | .11 | -.38^***^ | -.18^*^ | .00 |
| 4. Competence | -.09 | .00 | -.03 | - | .65^***^ | .71^***^ | -.07 | .00 | -.08 | .15^*^ | .06 | -.17^*^ |
| 5. Communion | .03 | -.00 | -.08 | .63^***^ | - | .48^***^ | -.02 | .06 | .05 | .18^*^ | -.04 | -.05 |
| 6. Agency | -.01 | .10 | -.03 | .74^***^ | .49^***^ | - | -.07 | -.02 | -.08 | .04 | .06 | -.19^*^ |
| 7. Attitude toward Minimun Vital Income | -.18^*^ | .25^***^ | .55^***^ | .10 | .08 | .13 | - | .03 | .13 | -.41^***^ | -.06 | .00 |
| 8. Covid economic threat | .06 | .05 | -.00 | .14 | .08 | .11 | .05 | - | .12 | -.05 | -.05 | -.12 |
| 9. Covid health threat | -.07 | .06 | .13 | -.06 | -.02 | -.07 | .09 | .14 | - | -.16^*^ | .00 | .03 |
| 10. Political ideology | .19^**^ | -.08 | -.42^***^ | .15^*^ | .19^*^ | .10 | -.30^***^ | -.07 | -.02 | - | .09 | .09 |
| 11. Subjective socioeconomic status | .04 | .06 | -.04 | .03 | .06 | -.00 | -.07 | -.11 | .01 | .19^**^ | - | .36^***^ |
| 12. Objective socioeconomic status | -.08 | -.03 | .18^*^ | -.20^**^ | -.16^*^ | -.20^**^ | -.06 | -.16^*^ | -.07 | -.05 | .38^***^ | - |

*Note.* correlations between variables in women in poverty condition (above the diagonal) and men in poverty condition (below the diagonal). ** *p* < .01 (2-tailed) * *p* < .05 (2-tailed.

**Supplement D4. Confirmatory Factor Analysis (CFA) for Stereotype Measure**

First, we test for multivariate normality in our data using the MVN package (Korkmaz et al., 2014). Henze-Zirkler's multivariate normality test (Henze & Zirkler, 1990) confirmed that data was not multivariate normal so, as stated in the manuscript, we used maximum likelihood estimation with robust standard errors, as we might get biased results with maximum likelihood estimations. Next, we conducted the CFA using lavaan package. We fitted a model with three factors (agency, communion, and competence) and indicators suggested an acceptable fit of the model: Chi-square value of the measurement model (*X*^2^) = 378.547; degrees of freedom = 167; comparative fit index (CFI) = .91; Tucker–Lewis index (TLI) = .89; root mean square error of approximation (RMSEA) = .06; Standardized Root Mean Square Residual (SRMR) = .06; Loglikelihood = -9276.91; Akaike Information Criteria (AIC) = 18639.83, Bayesian Information Criteria (BIC) = 18806.69.

**Supplement D5. Controls and Robustness Checks**

We did not find a significant interaction of experimental condition with participant gender on any of our dependent variables: individualistic attributions (*F*(1, 353) = 1.01, *p* = .31), communion scores (*F*(1, 353) = 0.83, *p* = .36), agency scores (F(1, 353) = 0.28, *p* = .60), competence scores (*F*(1, 353) = 0.06, *p* = .81), and attitudes toward social protection policies (*F*(1, 353) = 0.00, *p* = .98).

**References**

Hayes, A. F. (2017). *Introduction to mediation, moderation, and conditional process analysis: A regression-based approach*. Guilford publications.

Henze, N., & Zirkler, B. (1990). A class of invariant consistent tests for multivariate normality. *Communications in Statistics-Theory and Methods*, *19*(10), 3595-3617. <https://doi.org/10.1080/03610929008830400>

Pratto, F., Sidanius, J., Stallworth, L. M., & Malle, B. F. (1994). Social dominance orientation: A personality variable predicting social and political attitudes. *Journal of Personality and Social Psychology*, *67*(4), 741-763. [https://doi.org/10.1037/0022-3514.67.4.741](https://doi.apa.org/doi/10.1037/0022-3514.67.4.741)

Silván-Ferrero, M. D. P., & Bustillos, A. (2007). Adaptación de la Escala de Orientación a la Dominancia Social al castellano: Validación de la dominancia grupal y la oposición a la igualdad como factores subyacentes. *Revista de Psicología Social*, *22*(1), 3-15. <https://doi.org/10.1174/021347407779697485>

Vandello, J. A., Bosson, J. K., Cohen, D., Burnaford, R. M., & Weaver, J. R. (2008). Precarious manhood. *Journal of Personality and Social Psychology*, *95*(6), 1325–1339. <https://doi.org/10.1037/a0012453>
